# Potential Factors Conditioning the Compliance to Mandatory Face Covering in the Public Space Due to SARS-CoV-2 Pandemic

**DOI:** 10.3390/ijerph20010726

**Published:** 2022-12-30

**Authors:** Natalia Maja Józefacka, Emil Andrzej Karpiński, Barbara Superson, Mateusz Franciszek Kołek, Andrzej Robert Skrzypczak, Gabriela Kania

**Affiliations:** 1 Institute of Psychology, Pedagogical University of Krakow, Podchorążych 2, 30-084 Krakow, Poland; 2 Department of Tourism, Recreation & Ecology, Institute of Engineering and Environmental Protection, University of Warmia and Mazury, Oczapowskiego St. 5, 10-719 Olsztyn, Poland; 3 Diplomstudium Humanmedizin, Medizinische Universität Wien, Spitalgasse 23, 1090 Wien, Austria; 4 Students Scientific Club ControlUP, Institute of Psychology, Pedagogical University of Krakow, Podchorążych 2, 30-084 Krakow, Poland

**Keywords:** COVID-19, anxiety, social approval, reactions to crises, intergroup relations

## Abstract

Background: During the SARS-CoV-2 pandemic, we could observe different attitudes towards restrictive bans and orders. Aim: The research aimed to examine the potential psychological factors, such as generalized anxiety, fear of COVID-19 or social approval, related to the approach to mandatory face covering in public spaces. Methods: The web-assisted interviews survey was used among 202 participants, which included socio-demographical data, approach to face covering, the Generalized Anxiety Disorder Scale, the COVID-19 Anxiety Scale, and The Questionnaire of Social Approval. Result: The data showed a statistically significant correlation between compliance to the rule of face and nose covering vs. anxiety and compliance to the rule of face and nose covering vs. generalized anxiety. The results indicate differences between vaccinated and non-vaccinated people in the anxiety of COVID-19, generalized anxiety, and compliance with the rule of face and nose covering. Conclusions: People vaccinated has a higher level of anxiety and more often compliance with the rule of face covering. It is worth noting that an overly pronounced fear of COVID-19 could be a risk factor for mental health. More research about coping with anxiety in the group of vaccinated people is recommended.

## 1. Introduction

In Poland, the first case of COVID-19 was detected on 4 March 2020. The SARS-CoV-2 pandemic has spread worldwide, causing millions of people to be infected, and hundreds of thousands have lost their life. On 11 March 2020, the World Health Organization declared it a global pandemic. The rapidly spreading disease changed the functioning of societies all over the world. Moreover, the pandemic has become an intense challenge to the everyday life of not only public health but common citizens as well.

Legal regulations are an important instrument in the fight against the COVID-19 pandemic. Many restrictive bans and orders were introduced, which were associated with the debate in the media and the scientific community [1,2]. One such regulation introduced in Poland by the regulation of the Council of Ministers on 2 May 2020 was the obligation to cover the mouth and nose.

In light of legal regulations, these restrictions were discussed in the context of their legitimacy. There were numerous voices about limiting the constitutional freedoms and rights of an individual [3,4]. More knowledge about COVID-19 made it clear that obeying adopted prescripts is crucial to return to regular life. However, these requirements met some resistance. On the one hand, studies indicate wearing a mask to be a successful non-pharmacological method limit the spread of COVID-19 [5,6,7]. There were voices of scientists about the necessity to put on masks: “To simplify it, you can say that the lack of a mask can be fatal, but it is really a very simplification”,—said professor Włodzimierz Gut, a Polish virologist [8]. Ben Cowling, a professor of epidemiology and a mask researcher at the University of Hong Kong’s School of Public Health, said that people should wear the mask, not because it will prevent everyone from getting infected but because it will slow down transmission in the community a bit [9]. On the other hand, some studies suggested that only face coverings alone had no significant effect in interrupting the spread of respiratory viruses [10]. Furthermore, WHO said community masking could lead to a “false sense of security” and cause people to ignore other evidence-based measures like handwashing and self-isolation [9].

Notwithstanding, it is still a new area of research. According to the latest research in Poland, such as IBRiS for Rzeczpospolita [11] and Kantar for Gazeta Wyborcza [12], over 50% of the Polish are in favor of lowering restrictions due to the SARS-CoV-2 pandemic. In addition, a survey conducted by ARC Rynek i Opinia [13] indicates that 29% of Poles do not believe in the existence of COVID-19 at all. Particularly, citizens under the age of 35 are against any kind of restriction.

These unclear restrictions resulting from several sanitary and epidemic regulations, as well as numerous other stressors, influenced the psychosocial functioning of people. The unpredictability of the situation, as well as the feeling of danger, both social and health, created conditions that made it difficult to make rational decisions and adapt to top-down legal regulations.

It has been shown that the SARS-CoV-2 pandemic has a negative impact on mental health, including post-traumatic stress disorder, anxiety disorders, and depression [14]. The pandemic is conducive to the activation of numerous anxiety disorders in society. However, subjectless states of anxiety and a sense of threat, persistent all the time and not modified by the external situation, is a disorder that affects human behavioral functioning. Generalized Anxiety Disorder (GAD) is characterized by increased anxiety, which is also expressed by sleep disorders, excessive sweating, as well as somatic symptoms (muscle tension, increased heart rate and respiration, disorders of the digestive and genitourinary systems). In a pandemic, chronic anxiety is not surprising, especially among neurotic and highly sensitive people.

Nowadays, people are facing the constant fear of not only their own death but losing their relatives as well [15]. The fear of COVID-19, which has a potential significance for people’s compliance with the obligation to wear masks, was examined by the Fear of COVID-19 Scale (FCV-19S). It has been successfully validated questionnaire in many countries, e.g., Iran [16], Turkey [17,18], Italy [19], Israel [20], Saudi Arabia [21], Greece [22], United States [23], Japan [24] and Peru [25]. Studying the level of fear of COVID-19 in society has important practical implications: organizing appropriate support, monitoring the mental state of the society, and looking for the potential factors conditioning the compliance to mandatory face covering in the public space.

From the available scientific results, it appears that there is a need for further consideration of social behavior during a pandemic [14,15]. It is justified to make further observations on generalized anxiety, fear of COVID-19, and the need for social approval was carried out.

The main objective of the study was to examine the potential psychological factors conditioning compliance to mandatory face covering in the public space. The need for social approval, understood in this study as a personality trait and a tendency to present oneself in a falsely favorable light, seemed to be an important psychological factor that could influence compliance with the principle of wearing a mask. Therefore, we posed an exploratory research question, whether the relations between levels of these mentioned factors and obeying mask-wearing in the public area exist.

In our study, we also look at the level of generalized anxiety, that is, experiencing long-term, persistent general anxiety not related to any specific cause for at least six months. We assumed that there is a statistically significant relationship between the principle of covering the face and nose and the fear of COVID-19 and the level of generalized anxiety.

Furthermore, in our study, we analyzed the differences between vaccinated and unvaccinated people in terms of COVID-19 anxiety intensity, generalized anxiety disorder, and adherence to the principle of covering the mouth and nose.

## 2. Materials and Methods

### 2.1. Sample

This cross-sequential online survey evaluated potential factors conditioning face covering. Participants (*n* = 262) consist of the general population, recruited via Facebook and snowball sampling among respondents. Approximately 77% of the sample was female. The age of participants spanned 20 to 60 years (*M* = 23.05, *SD* = 6.80). For educational attainment, 4.4% of participants completed an elementary school, high school diploma, or general education diploma. Approximately 67.9% of participants attended college without completing a degree, whereas 27.8% of participants completed a college degree.

### 2.2. Measures

Participants were asked to answer three self-rating scales using their PC or tablet. They were also asked for information about their educational level, age, gender, and attitude toward face covering.

1. To evaluate the general level of anxiety, the Generalized Anxiety Disorder 7-item Scale (GAD-7) was used. The GAD-7 is a useful tool for screening for generalized anxiety disorder and assessing the severity of anxiety symptoms over the past two weeks. It has been shown to produce reliable and valid scores in community studies.
2. The Fear of COVID-19 was measured by FCV-19S [16,22]: FCV-19S is a brief instrument consisting of seven items, e.g., item 1, “I am most afraid of coronavirus-19”; item 5, “When watching news and stories about coronavirus-19 on social media, I become nervous or anxious.” Responders are asked to indicate their level of agreement with each of the seven statements using a five-item Likert type scale (1 = strongly disagree to 5 = strongly agree).
3. The Questionnaire of Social Approval (KAS) is designed to monitor the level of social approval, understood as a personality trait and a tendency to present oneself in a falsely favorable light. The questionnaire consists of 29 statements that require the respondent to answer “true” or “false”, describing behaviors and traits with explicit social approval or disapproval.
4. Vaccination, participants answer are they decided to vaccinate themselves or not.
5. Mask willingness is a scale designed to measure the willingness to cover the face and nose in various environments. Based on PCA outcome there was found two factors: indoor face covering and outdoor face covering.

### 2.3. Statistical Analysis

To define the connection between the anxiety of COVID-19, generalized anxiety, the need for social approval, and the adherence to the governmentally sanctioned rule of face and nose covering, statistical analysis with the use of R programming language [26] and JAMOVI open-source statistical software [27] was performed. Packages such as *readr* [28], *rstatix* [29], *psych* [30], *FactoMineR* [31], *facotextra* [32], *ggpubr* [33], *ggplot2* [34], and *GGally* [35] were loaded to the workspace to widen the range of possible analyses and data visualizations.

The reliability of all scales used in the study was assessed. To fulfill this purpose, Cronbach’s α, McDonald’s ω, and average Pearson’s correlation coefficient were calculated. Subsequently, principal component analysis (PCA) was performed only for the five questions about wearing a face covering to prevent COVID-19 transmission. According to the Kaiser criterion, two factors emerged and were used in the further analysis.

Descriptive statistics, as well as Shapiro-Wilk’s normality test, were calculated, and visual inspection of the data was conducted using histograms, kernel density estimation (KDE) plots, and quantile-quantile plots. As the sample size was fairly enough, the central limit theorem was used to deal with the sampling distribution’s normality assumption where possible.

The statistical indicator of wearing a face and nose coverage obtained by PCA analysis was then correlated with the anxiety of COVID-19, generalized anxiety, and the need for social approval. The results were presented in the form of a corrplot.

Differences between vaccinated and non-vaccinated people regarding their results on all subscales were assessed with Student’s *t*-test with Welch’s correction when necessary. The significance level of α = 0.05 was assumed.

## 3. Results

Cronbach’s α, as well as McDonald’s ω indicated a sufficient level of reliability in all but one subscale (Table 1). As the coefficient values for the second face and nose covering subscale were insufficient, it was removed from subsequent analyses, except for the calculation of descriptive statistics.

Before principal component analysis, the fulfillment of the Kaiser-Meier-Olkin criterion was assessed by calculating MSA (measure of sampling adequacy) coefficients. Their values for all variables were above 0.50, and overall MSA was equal to 0.66, which suggested that the dataset is adequate to further the dimension reduction procedure. Additionally, the Bartlett sphericity test indicated that the empirical correlation matrix differs statistically significantly from the identity matrix, K2(4) = 219.83, *p* < 0.001.

**Figure 1:**
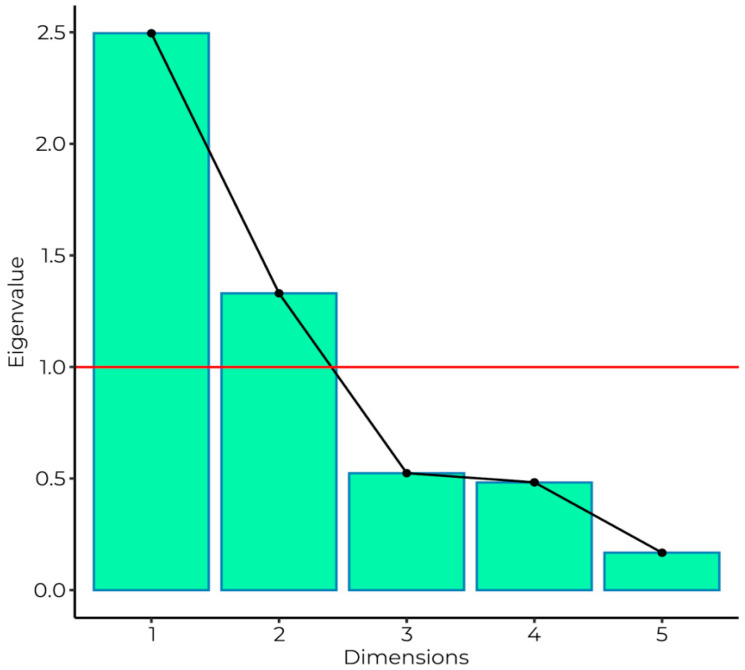
Scree plot illustrating the eigenvalues of subsequent principal components. The Kaiser criterion cutoff point was presented as the red line. Only two first dimensions fulfilled the formal criterion and were included in further analysis.

Principal component analysis was performed for five questions about wearing face and nose coverage in certain places. According to the Kaiser criterion (eigenvalue > 1), two principal components were chosen. Eigenvalues of principal components were presented on the scree plot, together with the cutoff point (Figure 1).

The first dimension consisted of three questions concerning wearing a face and nose coverage in closed public places (1st: shop/commercial place, 2nd: restaurants, 3rd: means of public transportation), whereas the second dimension regards the same issue in public open places (1st: park/playground, 2nd: forest). This conclusion was confirmed by the factor loadings presented in Table 2.

**Figure 2:**
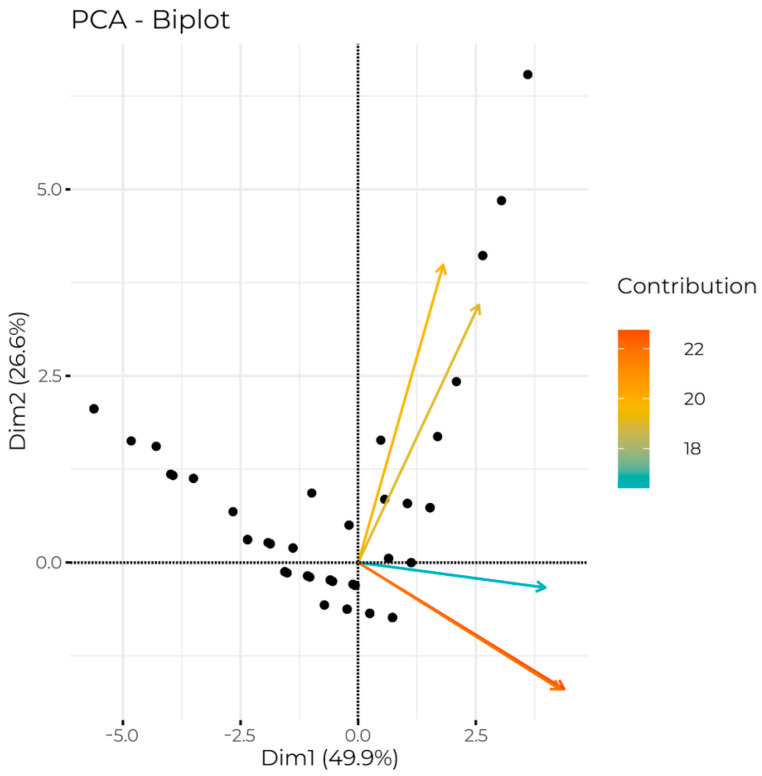
Biplot illustrating the placement of observations and variables on two-dimensional space. Variables (presented as arrows) were filled according to their contribution to the appropriate dimension. Observations were presented as black dots. The names of subsequent dimensions were accompanied by information about the percentage of explained variance in the output data.

**Figure 3:**
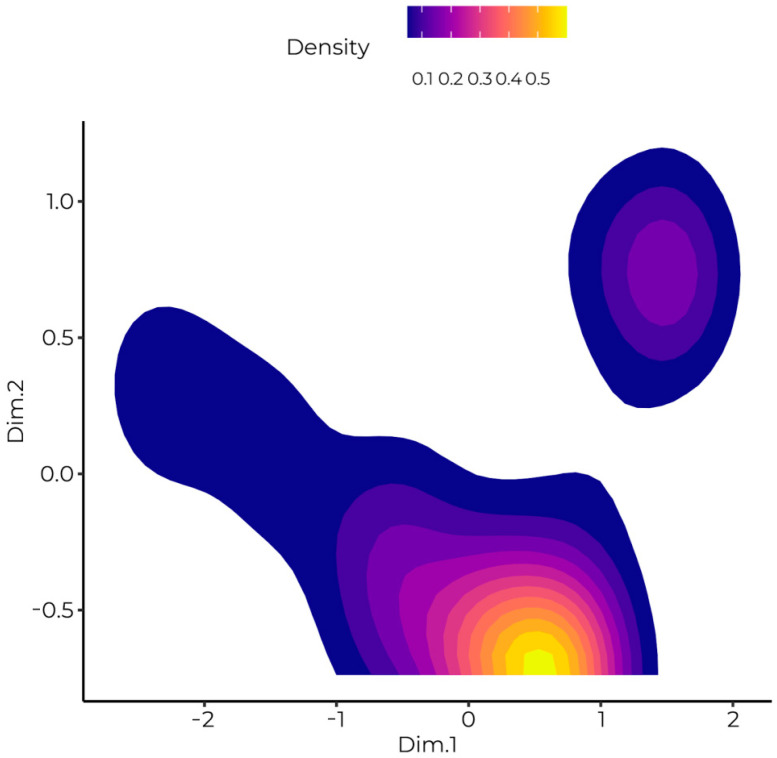
Two-dimensional density estimation for two principal components of face and nose covering. The highest density was observed near the axis crossing, and two extreme subgroups were detected.

Observations are divided clearly into two distinct extreme groups. The first complied with the rule of face and nose covering in closed and open public places, whereas the second group of people resigned from face and nose covering in closed places. Between them resides a big group centered around the intersection of the axes of the coordinate system, which represents people who sometimes forget to wear a face and nose covering in closed and open placesl. This phenomenon is presented in Figure 2. As the points in the plot are frequently overlapping, it is hard to estimate their density. Therefore additional illustration of density estimation is provided in Figure 3.

Descriptive statistics were calculated for age; both obtained PCA subscales, the anxiety of COVID-19, generalized anxiety, and the need for social acceptance. All results were approximately normally distributed, except for age and the second principal component (outdoor face and nose covering). The first principal component is also marked by higher skewness and kurtosis values; however, it does not exclude this factor from subsequent analyses, as the central limit theorem can be applied. The results are presented in Table 3.

**Figure 4:**
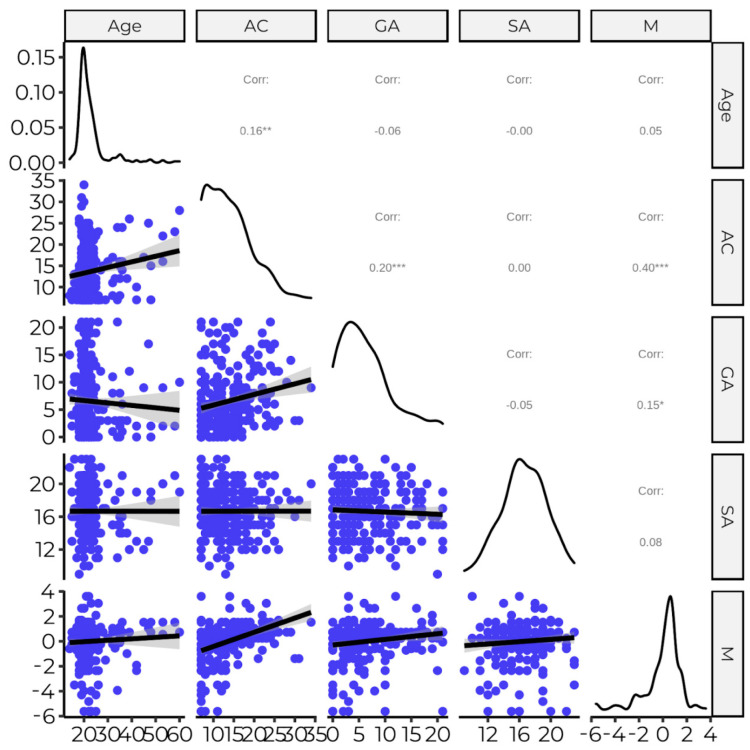
Corrplot for continuous variables. Above the main diagonal of the matrix, the correlation coefficient values and their significance was presented; at the main diagonal one dimensional density estimation of subsequent variables, and under the main diagonal two-dimensional scatterplots. Note AC—anxiety of COVID-19, GA—generalized anxiety, SA—the need for social acceptance, M—compliance to the rule of face and nose covering (indoors). Significance levels: * *p* < 0.050; ** *p* < 0.010; *** *p* < 0.001.

Analysis of Pearson’s r correlation coefficients revealed two statistically significant relationships with the first principal component from PCA (indoor face and nose covering). A moderate and positive correlation was observed between the anxiety of COVID-19 and compliance with the rules, whereas a positive and small correlation was assessed between generalized anxiety and face and nose covering (Figure 4).

It is worth mentioning that anxiety of COVID-19 and generalized anxiety also correlate, and the coefficient value can be interpreted as small and positive. Therefore it was highly recommended to calculate the partial correlation coefficient of anxiety of COVID-19 and face and mask covering, taking into consideration the effect of generalized anxiety.

The partial correlation coefficient was statistically significant at *p* < 0.001 and suggested a moderate positive relationship between Anxiety of Covid and Indoor Masks (r = 0.38).

The differences between vaccinated and not vaccinated people were assessed using Student’s t-tests. Their results were statistically significant for COVID-19 anxiety, generalized anxiety, and compliance with the rule of face and nose covering. Effect size estimates for COVID-19 anxiety and principal component suggested a moderate magnitude of differences, whereas for generalized anxiety it was rather small. Vaccinated people were marked by higher levels of all significant parameters. The results are presented in Table 4.

## 4. Discussion

In defining the research problem, we posed the main question: whether there are significant links between the intensity of pandemic factors and adherence to the principle of wearing a mask in public spaces. We hypothesized that there was a statistically significant relationship between the principle of covering the face and nose and COVID-19 anxiety and the level of generalized anxiety. In doing so, we considered the possibility of variation in the character of these relationships depending on access to vaccination.

Through the results, we confirmed a moderate positive correlation between COVID-19 anxiety and adherence to the rules, while a slight positive correlation was found between generalized anxiety and face and nose coverage. Furthermore, the results indicate statistically significant differences between vaccinated and non-vaccinated people in the anxiety of COVID-19 (AC), generalized anxiety (GA), and compliance with the rule of face and nose covering (M).

Our results agree with those obtained by McElfish et al. [36]. Participants in these studies (*n* = 754) reporting no fear (OR = 5.51; *p* < 0.001) and very little fear (OR = 1.95; *p* < 0.05) of COVID-19 had greater odds of COVID-19 vaccine hesitancy compared to people who were very afraid of COVID-19. In the literature [37,38], it has been found that knowing the level of fear of COVID-19 is an important point of psychological research during a pandemic due to educational and preventive activities.

An important aspect of our study was to identify whether there is a statistically significant difference between vaccinated and unvaccinated people in the context of factors that have the greatest impact on compliance with the principle of covering the face. The vaccinated persons were characterized by higher levels of generalized anxiety and fear of COVID-19. In contrast, the need for social approval was slightly greater in this group but it was not statistically different. Thus, regardless of adherence to vaccination, the need to conform to social expectations was equally important to all respondents. Thus, it should be considered an important determinant of life attitudes in the public space. According to Krings et al. [39], on research on the social psychology of group processes and intergroup relations, we know that the pandemic is a shared existential threat that profoundly affects our day-to-day lives. Moreover, this study suggests that there is a shift in social norms. However, this shifting of social norms is reliant on people actively changing their behaviors and conforming to new standards of social behavior [39]. Motivation to change a health behavior requires awareness of the personal risk associated with one’s actions [40]. Our reality has seen the appearance of the “new normality” in public life. On the one hand, it stems from anxiety, while on the other, it is the result of habituation and the acquisition of habits (frequent hand washing, wearing masks, using liquid disinfectants). It is worth noting that the constant stimulation of fear and, as a result, emotional reactions emotional have become an important factor in the adaptation process [41]. Thus, researchers argue that individual risk perception, or feeling personally at risk, effectively shapes health behaviors [42].

It has been shown that people who feel anxious about COVID-19 may be more likely to have a positive attitude toward getting vaccinated and acceptance of pandemic restrictions, including wearing a mask [43,44]. Szmyd et al. [45,46] showed that the fear of contracting COVID-19 and the fear of passing the disease to relatives were correlated with the willingness to vaccinate. Fisher et al. [47] found that willingness to vaccinate was highest among people who believe they may become infected with COVID-19 and become seriously ill. At the same time, this part of society showed a greater tendency to accept pandemic restrictions related to limiting social contact, restricting being in indoor public spaces, and wearing masks.

Acceptance and participation in vaccination, combined with greater acceptance of the use of protective masks, are more strongly associated with COVID-19 anxiety than generalized anxiety. In contrast, the group that did not take the vaccination was characterized by the lowest rates of acceptance of wearing masks and lower levels of declared fear of contracting the disease. Such characteristics should be interpreted as coronasceptic attitudes. Seddig’s et al. [48] research shows that positive attitudes toward getting vaccinated were supported by trust in science and fear of COVID-19, whereas negative attitudes were associated with acceptance of conspiracy theories and skepticism regarding vaccines in general.

It is noteworthy that even if coronascepticism and pandemic denial results in less direct anxiety about Covid and non-acceptance of vaccination and wearing the mask, the level of generalized anxiety is relatively high. This may be related to the other psychological factors causing generalized anxiety resulting from living in a pandemic situation, i.e., limited social contacts, restrictions in public places, interpersonal conflicts regarding the approach to the pandemic, etc. The impact of other factors on the level of generalized anxiety in a pandemic seems to be an interesting proposal for further retrospective research. Moreover, in our study, the perception of generalized anxiety within the vaccinated and unvaccinated groups is characterized by a relatively large variation, as indicated by the value of the standard deviation of this indicator. The background to such feelings and reactions seems to be very complex. People’s perceptions of the risk related to COVID-19 may be driven more significantly by their perception of the risk to the self and their loved ones than by their perceptions of the risk to others and society as a whole [49]. As such, appeals to personal risk and the risk to loved ones may be most effective in increasing risk perceptions for COVID-19 and, thereby, compliance with COVID-19 safety measures [50]. The current COVID-19 risk communication strategies frequently use messaging that calls upon large-scale pro-sociality, exhorting people to protect others by wearing masks and social distancing [51]. Messages such as “We’re all in this together” and “Wear a mask to protect others” appeal to innate impulses to help each other despite personal inconvenience [49].

## 5. Conclusions

The present study aimed to investigate whether factors such as the level of generalized anxiety, fear of COVID-19, or the need for social approval affect compliance with the obligation to cover the mouth and nose during a pandemic. We identified a significant correlation between compliance with the rule of face and nose covering vs. anxiety of COVID-19 and also vs. generalized anxiety. 

In addition, we have shown statistically significant differences between vaccinated and non-vaccinated people in the anxiety of COVID-19, generalized anxiety, and compliance with the rule of face and nose covering. Most people with a higher level of general anxiety and COVID-19 anxiety decided to follow the rules of face and nose covering indoors. A similar situation could be observed among vaccinated people. Thus, an important conclusion of our study is to confirm that anxiety is a major factor in pandemic behavior and is independent of social acceptance. 

The results contribute to the discussion related to the influence of psychological factors, i.e., anxiety on compliance with the principle of covering the mouth and nose during a pandemic. On the other hand, an overly pronounced fear of COVID-19, which is noticeable within the vaccination group, could be a risk factor for severe mental health issues due to the pandemic and may lead chronically to an inability to correctly and adaptively engage in preventive measures. Therefore, our results enlighten the need to improve risk communication strategies and promote a better preventive and therapeutic coping with several fears in the context of the pandemic.

## 6. Study Limitations

This study has some potential limitations. Firstly, the absence of control groups and information was obtained through self-reported questionnaires. Secondly, the present study was conducted on a heterogeneous, although not fully representative (for age) sample of Polish people. Thirdly, despite attempts to circulate widely on all possible social media platforms, wider participation was expected. Finally, as the sample was composed of Poles only, we cannot exclude that cultural diversity in the habit of face covering may influence results. However, considering the situation, this was the best possible methodology to reach the people to understand the psychological factor.

## Figures and Tables

**Table 1:** Results of reliability analysis.

Scale	Average Pearson’s Correlation	Alpha	Omega
FCV-19S	0.52	0.87 (0.85–0.89)	0.88 (0.83–0.97)
GAD-7	0.62	0.92 (0.90–0.93)	0.92 (0.91–0.98)
KAS	0.09	0.75 (0.70–0.79)	0.79 (0.75–0.98)
Mask	0.35	0.72 (0.66–0.77)	0.92 (0.87–1.00)
F1	0.65	0.81 (0.76–0.84)	0.86 (0.81–0.91)
F2	0.48	0.56 (0.44–0.65)	0.83 (0.65–1.00)

Note: Data for Alpha and Omega coefficients were presented as coefficient value (95% CI); 95% CI for α was calculated according to the Feldt’s method and for ω using bootstrap sampling (100 iterations); FCV-19S—COVID-19 anxiety, GAD-7—Generalized Anxiety Disorder, KAS—Questionnaire of Social Approval; Mask—the willingness of mask cover in different social situations, F1—factor 1 inside face and nose covering, F2—factor 2 outdoor face and nose covering.

**Table 2:** Factor loadings for the first two principal components of face and nose covering in various places.

Wearing a Mas	Dimension 1 (Closed Places)	Dimension 2 (Open Places)
Shop/commercial	0.87	−0.34
Restaurants	0.79	−0.07
Public transport	0.85	−0.34
Park/playground	0.51	0.69
Forest	0.36	0.79

**Table 3:** Descriptive statistics analysis.

Variable	*M*	*SD*	*Me*	*MAD*	*Min*	*Max*	*Skew.*	*Kurt.*	*W*	*p*
Age	22.77	6.81	21.00	2.97	14.00	60.00	2.93	9.81	0.66	<0.001
Mask	12.95	2.57	13.00	1.48	5.00	20.00	−0.72	1.51	0.93	<0.001
Anxiety COVID-19	13.65	5.54	13.00	5.93	7.00	34.00	0.83	0.34	0.93	<0.001
Anxiety generalised	6.54	5.24	5.00	4.45	0.00	21.00	0.95	0.33	0.91	<0.001
KAS	16.66	2.77	17.00	2.97	9.00	23.00	−0.08	−0.35	0.99	0.007
F1 indoor	0.00	1.58	0.25	0.72	−5.62	3.60	−1.46	2.97	0.86	<0.001
F2 outdoor	0.00	1.16	−0.63	0.17	−0.74	6.54	2.81	10.16	0.66	<0.001

Note: *M*—mean, *SD*—standard deviation, *Me*—median, *MAD*—median average deviation, *Min*—minimum, *Max*—maximum, *Skew.*—skewness, *Kurt.*—kurtosis, *W*—Shapiro-Wilk’s test statistic.

**Table 4:** Student’s *t*-tests results between vaccinated and non-vaccinated people.

Dependent Variable	Group		*t*	*p*	Cohen’s *d*	Magnitude
	Not Vaccinated (*n* = 68)	Vaccinated (*n* = 194)				
AC	11.38 ± 4.88	14.44 ± 5.55	−4.04	<0.001	−0.57	moderate
GA	5.40 ± 4.84	6.94 ± 5.32	−2.11	0.036	−0.30	small
SA	17.00 ± 3.09	16.54 ± 2.65	1.17	0.241	0.16	negligible
M	−0.81 ± 1.95	0.29 ± 1.33	−4.32	0.001	−0.66	moderate

Note: AC—anxiety of COVID-19, GA—generalized anxiety, SA—need for social acceptance, M—compliance to the rule of face and nose covering; data were presented as *M* ± *SD.*

## Data Availability

Not applicable.
